# PARIS: protocol for a prospective single arm, theory-based, group-based feasibility intervention study to increase Physical Activity and reduce sedentary behaviouR after barIatric Surgery

**DOI:** 10.1136/bmjopen-2021-051638

**Published:** 2021-12-22

**Authors:** Jennifer James, Wendy Hardeman, Helen Eborall, Mark Goodall, John Wilding

**Affiliations:** 1 Department of Cardiovascular and Metabolic Medicine, Institute of Life Course and Medical Sciences, University of Liverpool, Liverpool, UK; 2 School of Health Sciences, University of East Anglia, Norwich, UK; 3 Usher Institute, University of Edinburgh, Edinburgh, UK; 4 Primary care and Mental Health, Institute of Population Health, University of Liverpool, Liverpool, UK

**Keywords:** rehabilitation medicine, diabetes & endocrinology, public health

## Abstract

**Introduction:**

Increased physical activity and reduced sedentary behaviour can encourage favourable outcomes after bariatric surgery. However, there is a lack of evidence as to how to support patients with behaviour change. The aim of this study is to assess the feasibility of a physiotherapist led, online group-based behaviour change intervention to increase physical activity and reduce sedentary behaviour following bariatric surgery.

**Methods and analysis:**

Single arm feasibility study of a theory and evidence-based group behaviour change intervention based on the Behaviour Change Wheel and Theoretical Domains Framework using behaviour change techniques from the Behaviour Change Technique Taxonomy v1. The intervention has eight objectives and specifies behaviour change techniques that will be used to address each of these. Groups of up to eight participants who have had surgery within the previous 5 years will meet weekly over 6 weeks for up to 1½ hours. Groups will be held online led by a physiotherapist and supported by an intervention handbook. Feasibility study outcomes include: rate of recruitment, retention, intervention fidelity, participant engagement and acceptability. Secondary outcomes include: physical activity, sedentary behaviour, body composition, self-reported health status and will be analysed descriptively. Change in these outcomes will be used to calculate the sample size for a future evaluation study. Qualitative interviews will explore participants’ views of the intervention including its acceptability. Data will be analysed according to the constant comparative approach of grounded theory.

**Ethics and dissemination:**

This study has National Health Service Research Ethics Committee approval; Haydock 20/NW/0472. All participants will provide informed consent and can withdraw at any point. Findings will be disseminated through peer-reviewed journals, conference and clinical service presentations.

**Trial registration number:**

ISRCTN31524689.

**Strengths and limitations of this study**

- This is a pragmatic single arm study evaluating a theory and evidence-based group intervention, which was developed through evidence synthesis, review of relevant theory and qualitative research with the patient population, clinicians and commissioners.
- We have identified that it would be inappropriate to immediately progress to a full trial, and thus designed a single site feasibility study with our patient and public involvement group, that can answer study and intervention uncertainties.
- We have outlined our protocol for a group behaviour change intervention, delivered online to a clinically vulnerable patient group during a worldwide pandemic.

## Introduction

### Background and rationale

Obesity is defined as an excessive accumulation of adiposity that presents a risk to health, this is often indicated by a body mass index (BMI) greater than 30 kg/m².^1^ It is a non-communicable disease, and is associated with serious conditions including type 2 diabetes, cardiovascular disease, obstructive sleep apnoea and some cancers.^2^ Most recently the seriousness of obesity has been highlighted with data showing that people with obesity have an increased susceptibility to serious disease and mortality from COVID-19 when compared with the general population.^3 4^

The most effective treatment for severe obesity (BMI ≥35 with obesity-related complications or ≥40 without) is bariatric surgery. It rapidly induces significant weight loss and can improve complications of obesity such as type 2 diabetes.^5^ Surgery however is not a cure; weight can be regained so associated conditions can return,^6–8^ as a result there is increasing attention to long-term outcomes and how patients might be supported to optimise weight maintenance, physical function and to reduce complications of obesity in the longer term.^9^

Evidence from systematic reviews and meta-analyses shows that patients who are more physically active after bariatric surgery optimise their weight and fat mass loss and improved their fitness compared with those who do not.^10 11^ Evidence from a randomised control trial of 60 patients with a BMI ≥35 kg/m² randomised to either aerobic exercise, aerobic-strength exercises or control conditions found a greater reduction in weight, per cent body fat and fat mass in the intervention groups when compared with the control, which was statistically significant. Reduction in fat free mass was significantly lower in the aerobic-strength group when compared with the others.^12^ However, not everyone who undergoes bariatric surgery becomes more physically active. Evidence from a systematic review and meta-analysis identified that although self-report and objective physical activity (PA) increased at 12 months post surgery, this manifested as an increase in lower intensity PA and a reduction in moderate-to-vigorous intensity PA (MVPA).^13^ A longitudinal study compared levels of PA before surgery and 6 months thereafter using self-report and objective measures (accelerometery). Prior to surgery 10% of participants met the PA guidelines for health of 150 min/week MVPA according to both outcome tools. Post surgery this increased to 55% according to self-report but was not supported by accelerometer data which showed that only 5% of participants achieved this.^14^ Thus, it is likely that in post-surgical care, patients will report higher than actual levels of PA. Another behaviour of interest in this study is sedentary behaviour (SB). A transient increase in SB is associated with significant reductions in insulin sensitivity, decreased lower limb lean muscle mass and increased body fat,^15^ which is particularly relevant to this patient group who are already at risk of metabolic disease.

We have conducted a systematic review that has identified that to our knowledge, there have not been any studies that have been appropriately powered to detect a change in PA or SB in people who have undergone bariatric surgery. Our review included studies where an intervention was delivered either pre-bariatric or post-bariatric surgery as long as there were post-surgical measures of PA, SB or both.^16^ Much of the focus in the literature, is on the physiological effects of changing these target behaviours as opposed to how to facilitate changes. Where the effect of an intervention on these behaviours is considered it tends to be because they are part of a battery of outcome measures rather than being the primary outcome or behaviour of interest. Many interventions are poorly described in terms of the theory-base; behaviour change techniques are not always described according to the taxonomy and the rationale for their use is not clearly explained.^16^ This lack of detail makes replication of intervention conditions challenging. Furthermore, assessment of fidelity and review of engagement beyond recording the number of sessions attended makes it difficult to ascertain if the interventions have been delivered and received as intended.^17–19^ Thus, there is an evidence gap for interventions that aim to increase PA and reduce SB in patients who have undergone bariatric surgery that this study seeks to address.

To summarise, there is a need for high quality studies, based on theory and evidence that target PA and SB, must be accurately described, clearly communicated and transparently evaluated.

The proposed study is a feasibility study of a group, theory-based intervention delivered online using a widely available platform (Zoom) which will be supported by an intervention handbook.

The intervention development is robust; it is the result of evidence synthesis, review of relevant theory and qualitative research with the patient population, clinicians and commissioners. Evidence has been mapped against the Behaviour Change Wheel, which includes the COM-B model and the Theoretical Domains Framework.^20 21^ The intervention has eight objectives which will be met over the course of six intervention session, each of which will be 1–1½ hours in duration.

## Aim and objectives

The lack of evidence for interventions to increase PA and reduce SB means that it would be inappropriate to progress to a full-scale evaluation trial, and thus a feasibility study is warranted.

The aim of this study is to assess the feasibility of single arm, physiotherapist led, online group-based behaviour change intervention to increase PA and reduce SB following bariatric surgery. Once we have conducted this study we will be able to determine if the intervention could be evaluated in a future definitive study.

The objectives relate to uncertainties and are as follows:

1. To ascertain recruitment and retention to the intervention and study.
2. To investigate fidelity of the intervention, participant engagement with and acceptability of the intervention.
3. To test the feasibility of study outcome measures: questionnaires, accelerometery and dual energy X-ray absorptiometry (DEXA) scan.
4. To estimate the necessary sample size for a future evaluation study based on the chosen outcome measures.

### Design

This is a pragmatic feasibility study evaluating a group-based behaviour change intervention that seeks to support people who have had bariatric surgery to increase their levels of PA and reduce SB. Groups will comprise up to six participants, who will satisfy the inclusion and exclusion criteria below.

## Methods: participants, interventions and outcomes

### Patient and public involvement

A patient and public involvement (PPI) group, comprising three members who have previously undergone bariatric surgery, was recruited via Obesity UK at the start of development work and they have inputted into both the intervention and study design of this and previous studies, over approximately a 3-year period. They have helped to develop the research question, inputted into development work, helped to refine intervention objectives and have influenced study design. An example of this is their absolute rejection of the initial proposal to include randomisation to either the intervention or standard care group as they felt support to increase PA and reduce SB would be beneficial for anyone who had undergone bariatric surgery, thus randomisation was unacceptable.

In view of the strength of feeling from the PPI group, the current COVID-19 pandemic and the aims of this feasibility study, the team agreed with the PPI group not use a randomised design in this instance and to proceed with a single arm feasibility study. However, once we have the results of this study we will meet with the PPI group, explain the principle of equipoise and work with them on study design which will include determining an appropriate control condition. The contribution of the PPI group to this study has been invaluable, and they will continue to contribute through the trial management group (TMG) and in dissemination once the study is complete.

### Study setting

The study will be conducted at Liverpool University Hospitals NHS Foundation Trust (LUH), which is a large teaching hospital located in the City of Liverpool. It is a regional centre that provides specialist multidisciplinary medical and presurgical weight management services to patients in the North West of England, who have a BMI of 35 kg/m² with comorbid conditions or a BMI of 40 kg/m² without comorbid conditions, in accordance with previous specialist commissioning guidance.^22^ Patients must access a specialist weight management service as a prerequisite for referral for bariatric surgery funded by the National Health Service (NHS) in England, which is what this service provides.

### Sample size

This is a feasibility study and thus there is no formal sample size which has been calculated against a primary outcome measure. In future studies this is likely to be objectively measured PA or SB. A pragmatic sample size range has been set for 12–24 participants. Twelve is the minimum recommended sample size for a feasibility study^23^; successfully recruiting up to 24 participants would allow a more precise sample size calculation for a future trial.

### Eligibility criteria

Participants eligible for inclusion in this study must meet the following criteria at screening:

1. Bariatric surgery within the previous 5 years (eg, single anastomosis gastric bypass, gastric bypass, duodenal switch, sleeve gastrectomy).
2. Age 18+.
3. Able to provide written and informed consent.

Potential participants are ineligible if they have:

1. Gastric band or gastric balloon.
2. The presence of a medical condition that could compromise the participants safety if they inappropriately increased their PA, (eg, undertaking high intensity PA in the presence of poorly controlled diabetes, and acute or severe cardiac history requiring medical review). The nature of this intervention is such that closer supervision of these patients is not possible.
3. Bariatric surgery within the previous 8 weeks.
4. Any congenital cardiac condition.
5. Unable to converse in English.
6. Weight in excess of 204 kg (maximum safe working load of DEXA scanner).

### Recruitment procedures

A broad and pragmatic recruitment strategy has been proposed for this study.

1. Posters advising of the study will be displayed in patient areas of LUH.
2. Obesity UK, a UK charity that supports people living with obesity who may have had or be considering bariatric surgery, will advertise the study to their members online using the same posters.
3. A team member will review the database of patients referred for bariatric surgery by LUH within the previous 5 years. Patients will receive an information letter with a participant information sheet enclosed advising them to contact the principal investigator (PI) if they would like to know more information or to discuss taking part.
4. Patients who have previously contacted the PI to express their interest in PA research will be advised of the study via email with the participant information sheet attached.
5. The PI (first author) will attend online support groups attended by people who have undergone bariatric surgery.

All potential participants who contact the PI regarding this study will be provided with a participant information sheet and be given the time to consider this information and ask questions prior to providing informed consent, taken by the PI. At each visit participants will be asked to reconfirm their consent. If participants are unwilling or unable to provide their consent they will be withdrawn from the study.

### Intervention

The PARIS (Physical Activity and sedentary behaviouR after barIatric Surgery) intervention, is theory and evidence-based; informed by a systematic review^16^ to identify promising intervention components including behaviour change techniques, that are associated with interventions that were able to increase PA in this patient group. We also conducted a qualitative study with patients, clinicians and commissioners, which identified the meaning of PA for the three different stakeholder groups, influences on this and considerations for a future clinical intervention. The findings of these two pieces of research were synthesised resulting in eight intervention objectives. These objectives were then mapped against the Behaviour Change Wheel which includes the COM-B model, and against the Theoretical Domains Framework.^20 21^ The Theory and Techniques Tool^24^ informed the choice of behaviour change techniques (BCTs) from the Behaviour Change Technique Taxonomy (v1).^25^ BCTs could either be practically applied to the target behaviour, for example, BCT 1.1 goal setting (behaviour) where a goal is agreed and defined in terms of the behaviours (PA and SB) to be achieved,^25^ for example, a daily step goal or because they addressed an influence on the behaviour identified from the qualitative research, for example, BCT 5.3 information about health consequences (of being more physically active and less sedentary),^25^ for example, improved cardiovascular fitness.

Integral to this intervention are the handbooks; one for participants and one for facilitators, which will be available as a hard copy, electronically or both according to preference. The facilitator’s handbook mirrors that of participants’ but also includes additional information which would be superfluous for participants, such as the definitions of the BCTs used during intervention delivery.

The intervention will be delivered online using widely available software (Zoom), by an experienced Chartered Physiotherapist who is the PI on this study. The format of the sessions will be as follows: the handbooks will be used to guide the sessions and topics that are discussed. The PI will facilitate discussions between participants, providing answers only when necessary, for example, when discussing the consequences of PA, the PI will interject if participant discussions are not consistent with the evidence base. It is important to note that these are not education sessions per se, but rather sessions to support participants in making changes to their levels of PA and SB based on the evidence from our development work. Six sessions will be held over the course of 6–8 weeks, comprising up to eight people who have previously undergone bariatric surgery. Each session is expected to last between 1 and 1½ hours; it is anticipated that they will be held at the same time on the same day each week. All participants will receive the handbook, and will be encouraged to set their own goals to increase their PA and reduce their SB.

Intervention objectives are:

1. To explain the rationale for the intervention in the context of the observed levels of PA and SB in people who have undergone bariatric surgery.
2. To provide information about PA and SB; what it is, the consequences of it and how to appropriately pace and progress PA to avoid injury and delayed onset muscle soreness.
3. To discuss and provide information about the known consequences of increased PA and reduced SB and how this relates to the known consequences of bariatric surgery.
4. Provide information about the guidelines for PA and SB for different populations.
5. Provide information about behaviour change, using the Behaviour Change Wheel to explain the required conditions for this to take place.
6. Provide information about goal setting, developing implementation plans and how to appropriately evaluate behaviour change.
7. Provide information about the different types of motivation and how this can affect success.
8. To encourage participants to reflect and assess their perceived versus objectively measured levels of PA and SB.

BCTs used to meet these objectives are shown in table 1, with examples of how we think they might be used by participants.

**Table 1:** Intervention objectives and behaviour change techniques

BCT	Example of how the BCTs could be used in the intervention
1.1 Goal setting (behaviour)	Participants will be asked to set a goal defined in terms of the behaviour to be achieved. Eg, daily step goal.
1.2 Problem solving	Participants will be asked to review their goals and to assess any factors that made achieving the goal possible/impossible. Eg, was their goal of going to the gym more likely if they packed their exercise clothes the evening before?
1.3 Goal setting (outcome)	Participants will be asked to consider what outcome they want to be able to achieve as a result of their behaviour change. Eg, a 5 km charity fun run.
1.4 Action planning	Participants will be asked to plan in detail when they will perform the behaviour and it must include one of the following: context, frequency, duration or intensity. Eg, get off the bus one stop earlier on the days that they go straight home from work.
1.5 Review behavioural goal(s)	Behavioural goals will be reviewed with the participants. The goal may be modified, a different goal may be set or they may remain unchanged. Eg, if participants had an active transport goal, they may increase the number of days they plan to do this, or keep it the same. Conversely, they might set a new goal for active transport when they have more time, if this was not achieved.
1.7 Review outcome goal(s)	Outcome goals will be reviewed with participants; they may be changed; a new goal may be set or they may remain unchanged. Eg, participants might have achieved their outcome goal of jogging 5 km 1×/week, and may increase the frequency to 2×/week.
2.2 Feedback on behaviour	Participants will be asked to review their behavioural goals and discuss these in terms of form, frequency, duration and intensity. Advice and feedback will be offered to participants if necessary.
2.3 Self-monitoring of behaviour	Participants will be asked to keep a record of their physical activity behaviours, eg, steps/day which they can review. All participants will be provided with a Fitbit and there will be space in the handbooks for them to record information about their target behaviours.
2.6 Biofeedback	It will be explained to participants that feedback about the body—such as improved blood pressure can be helpful in maintaining behaviour change. Outcome measures in this study will also be used to provide feedback to participants (eg, body composition).
2.7 Feedback on outcome(s) of behaviour	The outcome of PA and SB will be discussed with participants, eg, if they report reduced shortness of breath or increased fitness after a period of increased PA behaviour. This will be explained in relation to their target behaviour, eg, a consequence of their increased PA is improved cardiovascular fitness.
3.1 Social support (unspecified)*	Participants will be encouraged to interact with each other as part of the intervention and we expect that they will encourage each other towards their goals. We acknowledge that being in a group does not necessarily mean that social support will be provided.
4.1 Instruction on how to perform a behaviour	Participants will be given advice on how to safely progress their PA to avoid delayed onset muscle soreness. Eg, to slowly and incrementally increase the distance or frequency of their walking goals.
5.1 Information about health consequences	Participants will be asked to discuss their perceptions of the consequences of increased PA and reduced SB from a health consequences perspective. If this information does not arise spontaneously, then it will be provided.
5.3 Information about social and environmental consequences	The wider consequences of performing the target behaviour(s) will be discussed with participants. Eg, they will be asked to discuss the consequences of increasing their steps (if this was their target behaviour), conversely, they will be asked to discuss the consequences of not performing their target behaviour(s).
5.6 Information about emotional consequences	Participants will be asked to consider the emotional consequences of performing their target behaviour, eg, if a participant’s target behaviour is to complete a weekly exercise class they will be promoted to consider how good they will feel after doing this.
6.1 Demonstration of the behaviour	Pictures of people with obesity being physically active will be used throughout the intervention handbook. These pictures will be sourced from the World Obesity Federation.
7.1 Prompts/cues	Participants will be advised that prompts and cues might be used to help behaviour change maintenance and to consider how they might use this. Eg, participants might choose to set an alarm on their phone or Fitbit after a period of sitting to prompt them to break up their SB.
8.1 Behavioural practice/ rehearsal	Participants will be advised that practicing their target behaviour can help with behaviour change maintenance even if it is not in the context of their goal, eg, using the bathroom upstairs at home, can be helpful if the behavioural goal is to use the stairs in the workplace.
8.7 Graded tasks	Participants target behaviour will be broken down into smaller incremental ‘chunks’, which will be made more difficult until the target behaviour is achieved, eg, if the target behaviour is to use the stairs, participants might initially choose to go down rather than up the stairs if this is easier.
9.1 Credible source	A physiotherapist will deliver the intervention. They were identified as 'the right healthcare professional' in the qualitative research
9.2 Pros and cons	Participants will be asked to consider and compare the pros and cons of changing their behaviour. Eg, they will be asked to consider the pros of being more PA, which might include improved fitness versus the cons, which might include less time for other activities.
10.7 Self-incentive	Participants will be asked to consider rewarding themselves in the future if they have attempted or made progress with their target behaviours. Eg, agree to a future reward if they try to walk more/achieve their step goal. Participants will be advised that this reward should not undermine their goal; eg, audiobooks might be preferable to physical books if the latter encourages increased SB and reduced PA.
10.9 Self-reward	Participants will be encouraged to praise themselves or use self-reward if they have attempted or made progress with their target behaviours. Eg, they will arrange for the receipt of a reward such as a new audiobook. Participants will be advised that this reward should not undermine their goal; eg, audiobooks might be preferable to physical books if the latter encourages increased SB and reduced PA.
12.5 Adding objects to the environment	Participants will be provided with a Fitbit and information on how to use this to facilitate their target behaviour. Eg, how to use the Fitbit to record steps, active minutes or set alarms to discourage prolonged sedentary behaviour, etc.
13.1 Identification of self as a role model	Participants will be asked to visualise themselves being physically active and less sedentary in the future.
13.2 Framing/reframing	Discussions about the consequences of increased PA and reduced SB will encourage participants towards health, and ‘non-scale victories’ rather than weight. Eg, being more PA can facilitate increased quality of life, and improved body composition which might translation into reduced clothes size, rather than weight loss.
13.5 Identify associated with changed behaviour	Participants will be encouraged to view themselves as individuals who are more physically active and less sedentary thereby constructing a new identify as someone who engages with their target behaviour(s).
15.1 Verbal persuasion about capability	At the end of every session, the group will be encouraged and confidence instilled in them, telling them that they have raised good discussion points and ideas of how to achieve their target behaviour(s).
15.3 Focus on past successes	Participants will be asked to consider their previous successes in changing their behaviour. Eg, think of a time when you were more physically active or less sedentary.
16.2 Imaginary reward	Participants will be asked to image a future version of themselves performing the target behaviour, eg, jogging 5 km and the positive feelings this would provoke.

BCT, behaviour change technique; PA, physical activity; SB, sedentary behaviour.

Full details of the intervention development work and resulting intervention will be reported in a separate manuscript currently in preparation.

### Outcomes

#### Feasibility outcomes

Uptake and retention to the study and intervention will be assessed through recruitment and screening logs which will capture the number of patients invited to participate, the number of support groups attended by the PI and those who contact the research team as a result of other recruitment strategies. An anonymised group register will be kept to record attendance at each of the sessions; and an end of study case report form will summarise study and intervention session attendance for each participant.

Intervention fidelity will be self-assessed by the PI, using audio recordings and a fidelity checklist that details the objectives of the session and BCTs to be used. All sessions of one participant group will be audio recorded for fidelity assessment. This will provide the opportunity for self-assessment and feedback in a timely manner, but might be considered a limitation as this introduces possible bias. We have already considered how we might mitigate against this in a future study, for example, by incorporating independent, timely peer-review feedback and recording every participant group so that conclusions regarding effectiveness reflect the intervention rather than suboptimal delivery of the intervention.

Engagement and acceptability of the intervention will be assessed through semi-structured interviews. Interviews will be conducted online via Zoom, or telephone and are expected to last up to 40 min. As recruitment rate and retention is unknown, the provisional target is for two participants from each group to be purposively sampled. We will aim to interview study ‘completers’, ‘non-completers’, those who have most recently had surgery, and those who are furthest out of surgery. We will also aim to interview those with the highest and lowest levels of PA and/or SB. All interviews will take place after final data collection period to avoid the interviews influencing participants’ levels of PA. An interview guide has been developed and focuses on participants’ experiences of the study and the intervention. The schedule has been reviewed and refined with the PPI group. It is expected that this will be further refined as the interviews progress, in line with the methodological approach of grounded theory according to Charmaz.^26^ Grounded theory has been chosen as the methodological approach as there are a number of uncertainties that we are keen to explore; such as acceptability of the study and intervention. This approach will allows us the flexibility to pursue topics as they arise, and thus our findings should be grounded in this primary qualitative research.

Study source data and case report forms will be used to assess concordance with the chosen outcome tools, for example, if accelerometers were worn and questionnaires completed as directed. Participants experience of these tools will also be explored in the semi-structured interview.

To avoid potentially confounding issues, ideally participants should not be recruited into other trials during their participation in PARIS. Where recruitment into another trial is considered to be appropriate and without having any detrimental effect to this study, this must first be discussed with the chief investigator (CI) JW.

#### Participant timeline

**Figure 1:**
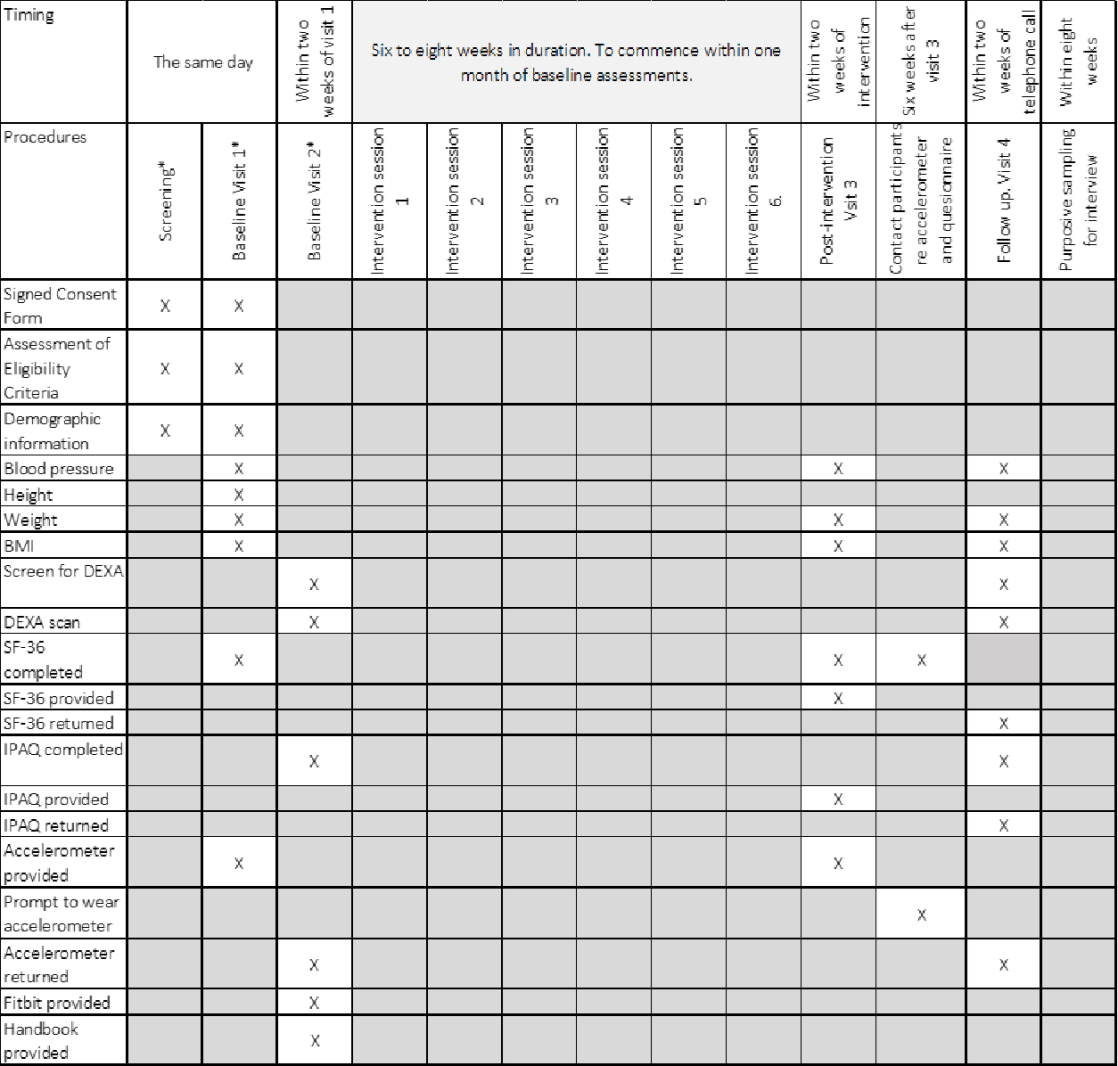
Schedule of events. BMI, body mass index; DEXA, dual energy X-ray absorptiometry; IPAQ, International Physical Activity Questionnaire; SF, short form.

There will be four study visits in total (see figure 1 schedule of events).

Visit one will be used to check eligibility, and to obtain informed consent, which will be taken by the PI. Participant demographics and some baseline measure will also be collected. Participants will be asked to self-report their health status using the SF-36 (RAND 36-Item Health Survey V.1.0)^27 28^ and to wear a wrist worn accelerometer (ActiGraph GT9X Link) for the next consecutive 7 days. Visit two will be within 2 weeks of the first; the accelerometer will be returned and participants will be asked to complete the International Physical Activity Questionnaire (IPAQ) (long form) and undergo a DEXA scan, thereby completing baseline data collection. The intervention will commence within 4 weeks of visit two.

Outcomes will be assessed again at visit three (post intervention) and for a final time at visit four (follow-up) approximately 6 weeks thereafter following a period of no intervention. Thus, all outcomes are assessed at three time points; preintervention (visits 1 and 2), post intervention (visit 3) and follow-up (visit 4).

PA and SB will be measured in two ways. Self-reported data will be collected using the validated IPAQ (long form)^29^ and objective PA and SB will be measured using ActiGraph GT9X Link, a water resistant, wrist worn accelerometer. Participants will be instructed to wear the accelerometer for seven full days (10 080 min), removing it as little as possible. In line with evidence from a systematic review that considered the cut points for PA classification in different age groups,^30^ we have chosen to use the Freedson *et al* 31 cut points as our population concerns adults. Self-reported health status will be measured using the SF-36 (RAND 36-Item Health Survey V.1.0)^27 28^ and DEXA will be used to measure body composition. DEXA is an accurate low risk procedure which will be carried out by a fully qualified healthcare professional in line with established departmental safety protocols.^32 33^

### Data management

All participants will be assigned a study identification number, which will be used on all paperwork and electronic databases. Consent forms will be stored separately from the research data; paperwork will be stored in locked cabinets and electronic databases will be password protected. All data will be accessible to the PI and CI during and after the study has completed, and will be available to sponsor and monitors as required.

### Statistical methods

Quantitative data will be analysed descriptively and this will be used to calculate the sample size that would be required for a fully powered trial. Analysis will occur after the study is complete, thus there will be no interim analysis of data. As this is a feasibility study, the rate of missing data itself will be an outcome. Due to the size of the study, there is limited capacity to assess patterns of missingness across treatment groups or known prognostic factors.

Qualitative data will be collected and analysed concurrently. Descriptive analysis will be used to identify codes and overarching categories that they construct. Interpretive analysis will then be used to identify subthemes and themes to which they belong allowing a greater understanding of the participants’ experience of the intervention and the acceptability of this. This process will be iterative informed by the constant comparative approach of grounded theory,^26^ with the aim of generating greater understanding of participants’ experiences of the intervention.

## Methods: monitoring

### Data monitoring

A TMG will be formed comprising the CI, PI, coauthors, PPI group, an external postdoctoral researcher and representative from the Liverpool Clinical Trials Unit. The TMG are responsible for monitoring the progress of the study, and will meet once ethics has been approved, once during the study and once more at the end. There will be additional meetings as and when required, and all meetings will be held online. The study will be open to audit from sponsor or their appointees as required.

### Harms

Adverse and serious adverse events are not expected as this study is of low risk. However, participants will be prompted to advise the research team if these occur and they will be recorded on the relevant documentation and databases, and will be followed-up as appropriate. Sponsor and LUH have the necessary insurance policies to cover any harm incurred by participants in this study.

### Withdrawal from the study

Participants may discontinue their involvement in the study for a number of reasons including, but not limited to:

1. Request by the participant.
2. Intercurrent illness.
3. Pregnancy if the participant wishes to withdraw or on the advice of their general practitioner or midwife.
4. Death.
5. Clinician led (any change in the participant’s condition that has been clinically reasoned to necessitate discontinuation of their participants, participant now meets the exclusion criterion).

Where possible, participants who are lost to follow-up will be contacted to collect outcome measures, even if they do not complete the intervention. The only exceptions to this are when consent is withdrawn or the participant is incapacitated and therefore unable to reconfirm their consent.

## Ethics and dissemination

All participants will provide written informed consent and will be free to withdraw from this study at any time without giving a reason.

Results of this study will be written up as part of a doctoral thesis and disseminated through publication in peer-reviewed journals, presented at professional conferences, shared via obesity and physiotherapy related meetings and via social media, for example, Twitter. Findings will also be shared with local clinical services, commissioning groups and patient support groups. A lay summary of results will be prepared with the PPI group and provided to study participants once the study is complete. Dissemination of study results is expected in 2022.

## Regulatory approval

This study has been approved by the NHS Research Ethics Committee (REC): Haydock 20/NW/0472. Any protocol modifications will be discussed with the TMG, reported to sponsor and the NHS REC.

## Study status

Recruitment began in March 2021 and the study is ongoing. It is realistic to suggest that the study might still be impacted by COVID-19, which may result in it being deemed inappropriate for participants to access the research site. However, PARIS has been developed in such a way that it minimises the potential impact from COVID-19. Site visits have been reduced, and the intervention has been designed to be delivered online. If there are further COVID-19 disruptions, it might be possible for the study design to adapt to reduce site visit frequency or duration further, for example, sending out questionnaires and accelerometer via recorded delivery to avoid participants coming to the study site. This would be discussed, as appropriate with the PPI group, trial management group, study sponsor and the REC.
